# How to Ensure Referral and Uptake for COPD Rehabilitation—Part 1: Disentangling Factors in the Cross-Sectorial Workflow of Patients with COPD to Understand why Most Patients are not Referred to Rehabilitation

**DOI:** 10.5334/ijic.5502

**Published:** 2021-03-02

**Authors:** Bettina Ravnborg Thude, Anette Brink, Michael Skriver Hansen, Lars Morsø

**Affiliations:** 1 Department of Medicine, Hospital Sønderjylland, Sønderborg, DK; 2 Health Department, Municipality of Sønderborg, Sønderborg, DK; 3 Open Patient data Explorative Network (OPEN), Region of Southern Denmark, DK; 4 Department of Clinical Research, University of Southern Denmark, Odense, DK

**Keywords:** COPD, cross-sectorial, FRAM, rehabilitation, referrals

## Abstract

**Introduction:**

Chronic obstructive lung disease (COPD) is one of the most serious and common chronic conditions. Patients having COPD can greatly benefit from rehabilitation initiatives. However, not all patients having COPD are referred to rehabilitation. Literature does not clearly explain why only some patients with COPD are referred to rehabilitation, and only very few successful solutions to address the complexity of cross-sectorial organisations are described. The overall objective of this research project is to ensure referral and uptake for COPD rehabilitation. We focus on detangling the processes in the cross-sectorial workflow of patients with COPD to understand why most patients are not referred to rehabilitation.

**Methods:**

Based on semi structured interviews and observations a FRAM analysis was conducted to map the referring routines from hospital to municipality.

**Results:**

We found that the hospital and the municipality have different understandings of what rehabilitation is, they use different words and hospital staff lack knowledge of offers at the municipality.

**Conclusion:**

The FRAM analysis was useful to detangle factors important to cross-sectorial collaboration and resulted in a series of focus areas that were disseminated at a workshop. The municipality and the hospital agreed to initiate activities to develop and coordinate the cross-sectorial relations.

## Introduction

### Problem Description

Chronic obstructive lung disease (COPD) is one of the most serious and common chronic conditions [1]. Patients with COPD have reduced quality of life, impaired health status and high mortality [2]. Most treatments have very limited effects, but the evidence that early rehabilitation after discharge supports the recovery of these patients is strong [3], and patients with COPD can greatly benefit from rehabilitation initiatives [4].

Therefore, it is important to offer patients rehabilitation as soon as possible after discharge or when seen in outpatient facilities. This is also stated in the newly revised Danish clinical guideline for the rehabilitation of patients with COPD. The guideline strongly recommends referral of patients to early rehabilitation after exacerbations requiring hospitalisation and initiation of rehabilitation when COPD is detected [5]. However, not all patients with COPD are referred to rehabilitation [6]. We have earlier tried to counteract challenges related to cross-sectorial handover and ability to initiate timely rehabilitation. In a pilot study setting, we tested the effect of targeted actions to increase referral, uptake and completion of early rehabilitation for COPD patients. Although we initiated activities to overcome barriers for healthcare providers and patients, we failed to increase the number of referrals significantly. However, patients who actually started rehabilitation had surprisingly high completion rates of individualised home-based tele-rehabilitation [7].

### Available Knowledge

The literature does not clearly explain why only some patients with COPD are referred to rehabilitation [8], and only very few successful solutions to address the complexity of cross-sectorial organisations are described [9]. More importantly, implementation is seldom guided by a theoretical framework to support a programme or an intervention in a given setting [10]. Studies show that thoroughly designed implementation of new interventions is as important as the content of the intervention itself [11]. In the light of known barriers for implementing substantial organisational transitions, there is a great need for studies that connect theory, guidelines and daily clinical reality.

### Objective

The overall objective of this research project is to ensure referral and uptake for COPD rehabilitation. In this part of the study, we focus on detangling the processes in the cross-sectorial workflow of patients with COPD to understand why most patients are not referred to rehabilitation, and use this knowledge to improve the development, implementation and evaluation of the patient pathway across healthcare sectors.

### Rationale

In 2016, the municipality and the hospital involved in this study formed a project group. The purpose of the group was to create improved patient pathways and to ensure that more patients were referred to rehabilitation services in the municipality. In 2017, the municipality and the hospital department requested researchers involved in the project to conduct an analysis of how the patient pathways were operating and under which conditions the collaboration between the municipality and the hospital could be optimised. Based on the theory of work-as-done (WAD) and work-as-imagined (WAI) [12], the primary focus was to understand how work was carried out on a daily basis—work-as-done. This is relevant in healthcare, as healthcare can be defined as a complex system, where humans adapt to the demands of their surroundings [13,14,15,16].

In a complex system, ‘work-as-done’ will always be different from what is planned or agreed on – ‘work-as-imagined’ - because it is not possible to describe and plan the system in too much detail [17, pp 31–39]. Therefore, staff have to adjust to the situation to handle their tasks. Consequently, the intention was to carry out an analysis of working routines at the hospital and the municipality regarding patients with COPD and the referral of these patients from the hospital to the municipality. Furthermore, the intention was to understand the organisational and structural conditions underlying the working routines.

A functional resonance analysis method (FRAM) [17] was chosen to analyse the working routines. The FRAM is a methodology to analyse everyday activities to understand the routines of everyday. It is developed to analyse complex systems and can provide an understanding for why things often go well but sometimes can go wrong [17]. The FRAM illustrates the interactions in complex systems and it can be used to model intractable complex systems [18]. Earlier, the FRAM has shown promise in being able to inform an intervention [19] and it has been used in a number of different complex systems such as aviation, maritime operations, railway and healthcare. The method can be used to analyse the resilience of a system, making it possible to describe interactions of various functions in a system and understand how people and equipment work together [20]. As the purpose of the analysis is to understand daily practice to target which interventions should be addressed to achieve a higher referral rate, we found the FRAM to be a suitable method. In contrary to Root Cause Analysis, the Domino Model, TRIPOD, The AcciMap approach, STAMP and Bow-Tie, it is not exclusively a risk analysis method but can be used for task analysis, system design etc. Furthermore, it produces a model of the activity instead of using a model developed beforehand [21]. The FRAM has earlier been described as a young method, that needed more development [18]. However, that was almost a decade ago, and the method has been used in many industries and case studies since then and is relevant in understanding the work as it is done [22].

In the FRAM, the working routines are mapped as functions that are connected. Each function has six aspects:

- Input (I) starts the function
- Preconditions (P) are conditions that have to exist before a function can start
- Resources (R) are what is needed while the function is carried out to achieve a good output
- Control (C) is what controls or regulates the function
- Time (T) is how time affects the function
- Output (O) is the result of the function.

**Figure 1:**
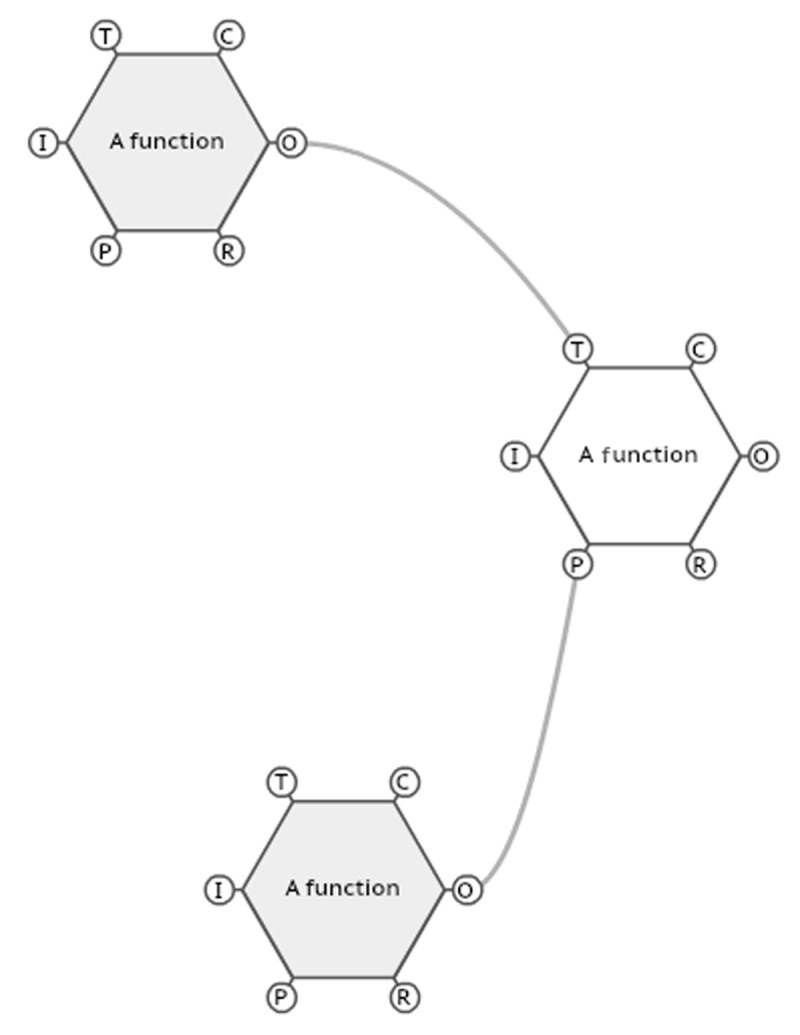
FRAM hexagons in connection.

The first five aspects derive from the output of other functions and are influenced by other functions. The functions are visualised as a hexagon, and the connections are shown as lines connecting the hexagons [17, pp 43–46], as shown in ***Figure 1***.

The figure shows different functions, and in some ways, it looks like any other task analysis that divides the process in sequences. However, during the questioning, and when drawing the model focus is on the interconnectedness and how one function interrelates with the other functions, and the lines in the figure show how the functions are connected. The visualisation is used to operationalise and manage the outcome but as any other model, it will only be a reduced picture of reality.

## Methods

### Context

The setting is the Danish Healthcare System, consisting of the hospital, general practitioners (GPs) and municipalities. Treatment is provided by the GPs and at the hospital. Both are financed by taxes and, therefore, free of charge for the individual patient. The municipality provides rehabilitation and care. Rehabilitation initiatives can vary from municipality to municipality but are also free of charge. The rehabilitation initiatives have gradually been moved from the hospital setting to the municipality, and from January 2018, the municipality will manage all rehabilitation and prevention initiatives for patients with COPD. In this particular case, a department for internal medicine at a hospital and the municipality where the hospital is situated are used as empirical information. Focus is on rehabilitation in the municipality after hospitalization, which in Denmark does not include care provided by the GPs, thus explaining why it is only the workflow at the hospital, and the municipality that is analysed.

The clinical guidelines in Denmark recommend rehabilitation when patients with COPD are discharged from hospital. It is a national goal that all patients with COPD should be informed of the rehabilitation services on offer in the municipality [23]. The national clinical guideline is considered a “golden guideline for what the clinical intervention should contain for patients with COPD”. In the same way, the Regional Collaboration Program for people with COPD [24] could be considered a golden guideline. We use these guidelines to define how healthcare services were planned and as an expression for WAI.

The wards at the hospital have 70 beds and 7700 inpatients a year; the municipality has 75,500 inhabitants.

### Data Collection and Ethics

Data for the analysis were collected through documents, observation of work-as-done and interviews of staff involved in the workflow. We conducted observations and interviews at a medium-sized hospital in the southern region of Denmark and at the municipality in adjunction to the hospital. Relevant staff and involved patients at the ward gave consent for participation.

By request, the Scientific Ethics Committee of Southern Denmark stated that no ethical approval was needed for this project. The study has been approved by the Danish Data Protection Agency (file no. 15/3321 and 19/3451). All rules of storage of personal information are met according to the Danish Data Protection Agency. Data will only be reported in anonymous form. Data management will be conducted according to the rules of the region of Southern Denmark at the department of OPEN (Open Patient data Explorative Network).

### Intervention and Analysis

The FRAM is based on observations and semi-structured interviews [25]. To conduct the FRAM, we first read relevant guidelines and instructions to understand work-as-imagined. After that, three FRAM experts each spent 1 day at different wards of the hospital observing the staff and conducting seven semi-structured interviews [26] with staff (physicians, nurses and therapists) and three semi-structured interviews with patients. The three FRAM experts also each spent 1 day in the municipality observing and interviewing five clinicians, the leader of the COPD rehabilitation unit, one district nurse who visits patients in their homes, and a nurse specialised in COPD treatment and care. In all, 15 interviews were conducted and we had all together six whole days of observations divided amongst the municipality and the hospital.

The semi-structured interviews were designed according to the aspects in the FRAM functions, and respondents were asked about input, preconditions, resources, time, control and output in a way that made sense to them.

The four researchers involved in the project at that time coded the data from the interviews and observation notes and categorised them in working functions according to the six aspects in each function. It was performed as a group where all participants had the written transcripts of the interviews; from these, the coding was derived. If there were discrepancies, these were discussed to consensus. We used the FRAM Model Visualizer software to build and display the FRAM model (REF *http://functionalresonance.com/FMV/index.html*).

Although it is recommended, we did not have the transcribed interviews validated by the participants, but the FRAM model itself, based on the interviews, was validated by presentation at a workshop where most interview participants attended together with staff that had not been interviewed. The validation of the FRAM model contributes to a strong method, as staff that had not been part of the analysis had the opportunity to make adjustments and the model came a step closer to showing a picture of the work as it is done.

## Results

**Figure 2:**
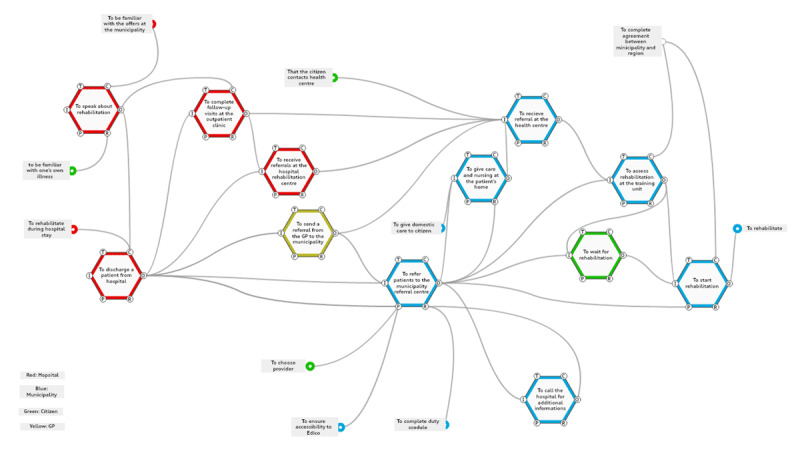
Results of the FRAM analysis.

***Figure 2*** shows the results of the conducted FRAM analysis of WAD of the referring routines from hospital to municipality.

The figure consists of 12 linked functions described in the following.

### “To discharge a patient from hospital”

The function has various outputs, depending on the situation of the patient. The patient can be either discharged to a follow-up visit at the outpatient clinic or treatment can be finished with no need for further rehabilitation, and a discharge summary is sent to the GP. In the event that the patient on discharge needs further rehabilitation or educational initiatives from the municipality, a notification will be sent to the municipality referral centre.

These are the outputs of the function. For some patients, all outputs will be activated whereas for others only some will be activated.

The national guideline [23], WAI, recommends that all COPD patients that are discharged from hospital are offered rehabilitation in the municipality. Therefore, we see a deviation from WAI and WAD.

### “To speak about rehabilitation”

Speaking about rehabilitation controls how the discharge of the patient and the follow-up visits at the outpatient clinic are handled.

During observations at the hospital, we found that the nurses in some cases did not mention rehabilitation or what the patient could gain from rehabilitation, even though it was relevant for the patient. By later questioning the nurses concerning that issue, one nurse stated that “maybe we have a much too limited understanding of what rehabilitation is.” The national guideline [23] argues that all patients with COPD should have rehabilitation, which is why the hospital should refer the patient to rehabilitation in the municipality, and again we see a difference from WAI and WAD.

### “To complete follow-up visits at the outpatient clinic”

Patients who are discharged with use of extra oxygen are scheduled for an outpatient follow-up visit twice a year. The patients that are incapable of going to the hospital can have a home-visit from a nurse from the hospital’s outpatient clinic.

The nurses from the outpatient clinic mentioned that approximately 90% of the patients using extra oxygen had rejected an offer of rehabilitation, many of whom regretted this after a period of 3 months. The nurses in the outpatient clinic can refer these patients to COPD rehabilitation at the municipality. However, the nurses in the hospital’s outpatient clinic do not know what the municipality can offer in terms of concrete rehabilitation initiatives.

The follow-up visits can be an input to the function “To accept the patient at the rehabilitation clinic at the hospital” and/or to the function “To receive a referral to the rehabilitation centre at the municipality”.

### “To send a referral from the GP to the municipality”

After the patient is discharged from hospital, the GP can send a referral to rehabilitation at the municipality, or a referral to the municipality referral centre, asking them to consider which kind of rehabilitation, nursing, training or prevention is needed.

### “To refer patients to the municipality referral centre”

The function at the municipality referral centre is activated by a notification from the hospital discharging the patient or a referral from the GP. The preconditions for the task to be carried out are that a plan for rehabilitation has been made by the hospital, that the patient has chosen a supplier of the health service and that the municipality has access to the patient’s electronic journal.

When lacking information, the municipality referral centre will have to contact the hospital, asking for further information on the patient. Some wards at the hospital have nurses dedicated to handling the discharge of patients, concerning contact to the municipality and relatives. The municipality experiences the discharging nurses as a big help, as “they know what kind of information the municipality needs”. Furthermore, the staff at the municipality referral-centre state, that it is an advantage knowing the discharging nurses at the hospital—“all in all it makes everything in the collaboration easier”.

There are several outputs from the function. The municipality referral centre can decide that the patient should have nursing help from the municipality at home and can define the kind of help that is needed. They can refer to the training unit at the municipality for assessing the patient’s needs. There can be a waiting list for rehabilitation, and in that case, the patient will have to wait for rehabilitation. Finally, they can initiate a rehabilitation plan, which is a precondition for starting the rehabilitation at the municipality.

The municipality referral centre has experienced on multiple occasions that the hospital promised patients a certain health service in the municipality that does not always match the services the municipality can provide. This causes frustration to patients and relatives, and it is time consuming for the municipality to handle the frustration and correct misunderstandings.

### “To give care and nursing at the patient’s home”

The district nurses at the municipality provide care to the patients in their home. When a patient is discharged from hospital, the municipality referral centre notifies the home-care nurses. The homecare nurse visits the patient on the day of discharge if they are notified that help is needed. One nurse stated that “if a citizen is afraid, it can spread to the family and relatives, who choose to call 911 [112 = Danish emergency]”. To avoid an unnecessary re-admission to the hospital, the homecare nurses see this as important to prevent. Furthermore, the homecare nurses can contact a team of acute nurses at the municipality if they need further help. The acute team can also be contacted by the hospital or GP if they find that a patient needs acute care and treatment.

The homecare nurses sometimes by coincidence discover that a patient has COPD if they see an inhalator at the patient’s home or observe COPD medicine on the medication list of a patient. If they do so, they can initiate rehabilitation of the patient at the municipality rehabilitation centre.

The nurses stated that patients lack knowledge of own illness and how to handle it. They believe that patients should be given more information on their illness and be informed about the benefits of rehabilitation much earlier, and that this may motivate the patients to accept an offer of rehabilitation.

### “To receive referrals at the municipality rehabilitation centre”

The rehabilitation centre at the municipality offers rehabilitation for patients with COPD. The rehabilitation includes classes in handling illness and exercising. The patients can come back and attend the classes several times. The patients can contact the rehabilitation centre on their own and can attend the same classes as if referred from the hospital, GP or homecare nurses. In approximately 50% of the cases, the patient contacts the rehabilitation unit directly.

The rehabilitation starts when a nurse from the centre contacts the patient for a talk to obtain a picture of the needs of the patient and clarify what kind of rehabilitation will benefit the patient.

We asked three patients about their experiences with COPD rehabilitation. None of them had received any information about the rehabilitation offers at the municipality. One patient stated that the GP did not inform them about rehabilitation but that relatives “found something on the internet”. Another patient with a 3 year history of COPD and who is now on oxygen treatment explained that they spend most days lying in bed. The patient has never received an offer of rehabilitation but has practical help at home. The patient explained that, “I have nothing to eat. They place the food in front of me and then they are gone. I need help for eating, but it cannot be handled in 12 minutes”.

***Figure 2*** shows how working routines are connected.

In addition to the FRAM model, the interviews also showed a different understanding of what rehabilitation is and a different use of words for the same work from municipality to hospital.

Based on the differences between hospital and municipality that were illustrated in the interviews and based on the FRAM model and the deviation between WAI and WAD, the following topics were identified in the analysis for further attention:

- The hospital and the municipality have different understandings of what rehabilitation is. At the municipality, the concept covers much more than when it is employed at the hospital, where they mostly consider it as training.
- The hospital and the municipality use different words, such as diagnosis versus level of function.
- Staff at the hospital lack knowledge of the offers at the municipality and methods of referral.
- The referral in the municipality depends on the information from the hospital. The referrals occasionally lack information, and when that occurs, municipality staff have to contact the hospital.
- Approximately only 10% of patients discharged from the hospital have a rehabilitation plan and continue directly in rehabilitation. For the remaining 90%, it is coincidental.

Based on the FRAM analysis, interviews and previous experiences [7], the hospital and municipality arranged a 1-day workshop. Staff from both sectors and patients/citizens were represented to discuss the topics that were pointed out in the analysis and to give input on how to reduce the barriers for creating a smooth patient pathway. The discussions at the workshop resulted in a wish from all sides to gain more knowledge and understanding of the work in the opposite health service sector, improved relations and communication across healthcare sectors, and more focus on how to motivate the patients/citizens to accept the rehabilitation offers in the municipality.

The results from the workshop were subsequently discussed in a cross-sectorial project group. The municipality and the hospital agreed to initiate a series of activities to develop and coordinate the cross-sectorial relations with the aim of ensuring an increase in referrals to prevention initiatives at the municipality.

## Discussion

The topics that were brought into the workshop as a result of the FRAM analysis helped to disentangle which factors in the cross-sectorial workflow of patients with COPD were of importance in order to understand why most patients are not referred to rehabilitation. At the same time, the workshop revealed a request for more knowledge and collaboration across healthcare boundaries. The participants explicitly communicated this request by stating differences in the understanding of the concept of rehabilitation, and different language and approaches in terms of diagnosis and functionality. The participants also stated that the lack of knowledge and information across healthcare sectors complicates collaboration across healthcare. Overall, the participants stated that cross-sectorial collaboration is easier if you know who is “in the other end of the line”, and they suggested activities to develop and coordinate the cross-sectoral relations.

The study was conducted in Denmark, and we argue that it has its relevance in all healthcare settings where two different parties handle hospitalisation and rehabilitation. Furthermore, the study is relevant for other diagnosis groups, where patients can benefit from rehabilitation.

The study was conducted at one hospital department and one municipality, which can be seen as a weakness. We expect that inclusion of more hospitals and municipalities would have resulted in more and/or other functions in the FRAM and different connections between the functions. However, we still find this study relevant and the context generalizable to other settings and nationalities.

Our findings are in line with research that shows that interdisciplinary cooperation is essential because healthcare is a complex system that one single practitioner cannot handle alone [27]. Additionally, research by Gittell [28] has shown that it is not sufficient to have good and dedicated staff. Because of the complex system, staff have to cooperate and coordinate—whereby a certain level of relational coordination is needed.

Relational coordination covers two themes: that staff have (i) good relations, meaning shared goals, shared knowledge and mutual respect, and (ii) good communication, meaning frequent communication, timely communication, accurate communication and problem-solving communication [29, pp 13–23]. An increased level of relational coordination between professionals in other settings has been shown to produce better clinical results and increased patient satisfaction [29, pp 25–47]. Even though the research was performed at American hospitals, it seems plausible that the findings are valid and can be applied to a Danish setting where healthcare also is complex.

Braithwaite argues that we have to accept a certain degree of uncertainty in healthcare due to complexity. We must take non-linearity and inconsistency into account, and in a complex system, we cannot expect that a certain intervention automatically leads to a predicted outcome because unpredicted circumstances within the system interfere with and affect intended actions. Methods to capture this complexity must be applied to develop quality in healthcare [30]. By conducting FRAM analysis in collaboration with patients and clinicians who know how “work is done”, and afterwards having these procedures verified, we are able to capture some of the complexity in the patient’s healthcare pathway and obtain insights into the unintended “stuff” that hinders planned procedures. The FRAM allows us to compare “work-as-done” with “work-as-imagined” to analyse the interference of regulations and protocols dispatched from daily clinical routines, the fragmentation of services, and the mismatches between workforce supply and system demand. Therefore, it is plausible that the FRAM captures the complexity of the daily working routines and provides a deeper understanding of specific factors affecting the cross-sectorial workflow.

These factors might not have been revealed using other methodological frameworks, such as lean production [31], the domino theory [32] and root cause analysis [33]. Lean was introduced in the 1950s for the car industry in Japan [34] and was used in a time and in an industry that was much more linear and predictable than the healthcare sector is today. The domino theory was developed in the 1940s and is similar to failure modes and effects analysis (FMEA), fault tree analysis (FTA) and event tree analysis, a model that divides processes into sequences [35]. However, according to Ellis and Herbert 2011, complex systems must be understood as a whole and cannot be broken down into pieces [36]. Root cause analysis (RCA) was also developed for the car industry [30] and not for a complex system, such as today’s healthcare. Compared with the RCA-MTO method, the FRAM has been shown to deliver better clarification and understanding of complex situations [37, pp 153–165].

Consequently, we found it relevant to use the FRAM, as we were analysing a complex system.

## Conclusion

The FRAM analysis was useful to disentangle factors important to cross-sectorial collaboration and resulted in a series of focus areas that were disseminated at a workshop. The workshop resulted in a request for more knowledge and collaboration across healthcare boundaries, and a common understanding, language and approach towards the concept of rehabilitation, diagnosis and functionality. To meet the workshop results, the municipality and the hospital agreed to initiate activities to develop and coordinate the cross-sectorial relations to ensure that the hospital would refer more patients to rehabilitation and prevention initiatives at the municipality.
